# Mitochondrial Metabolic Reprogramming in Glioma: Mechanisms of Tumorigenesis and Implications for Clinical Therapy

**DOI:** 10.1002/cns.71180

**Published:** 2026-09-28

**Authors:** Liangqi Jiang, Mingrui Li, Zhen Li, Qing Liu, Yang Li

**Affiliations:** ^1^ Department of Neurosurgery Xiangya Hospital, Central South University Changsha Hunan China; ^2^ Clinical Research Center for Skull Base Surgery and Neurooncology in Hunan Province Changsha Hunan China

**Keywords:** glioma, lipid metabolism, mitochondrial metabolic reprogramming, mitochondrial metabolism, molecular targeted therapy, protein metabolism

## Abstract

**Background:**

Gliomas are metabolically heterogeneous tumors in which mitochondria coordinate bioenergetics, biosynthesis, redox homeostasis, stress adaptation, and treatment responses. This review examines mitochondrial dependencies across glioma subtypes and cell states.

**Methods:**

We synthesized evidence on mitochondrial integration of glucose, amino acid and protein, lipid, and nucleotide metabolism, together with mitochondrial genetics, signaling, intercellular transfer, and barriers to therapeutic translation in gliomas.

**Results:**

Glioma glucose metabolism does not follow a uniform Warburg phenotype. IDH‐mutant gliomas exhibit D‐2‐hydroxyglutarate‐driven metabolic and epigenetic remodeling, whereas IDH‐wild‐type glioblastomas contain glycolytic, oxidative phosphorylation‐enriched, and adaptable stem‐like states. Mitochondrial proteostasis links protein import and translation with PI3K/AKT/mTOR signaling, the ubiquitin–proteasome system, autophagy, and mitophagy. Lipid synthesis, storage, fatty acid oxidation, and cardiolipin homeostasis support metabolic adaptation. Electron transport, aspartate availability, redox balance, and dihydroorotate dehydrogenase connect mitochondria with nucleotide synthesis, DNA repair, and treatment resistance. Mitochondrial DNA alterations, mitonuclear signaling, and intercellular mitochondrial transfer further influence respiratory adaptation and tumorigenicity. Metabolic compensation and intratumoral heterogeneity limit single‐target therapies, whereas clinical evidence supports genotype‐directed intervention, exemplified by vorasidenib.

**Conclusions:**

Effective mitochondrial targeting requires biomarkers that match metabolic dependencies to molecular subtypes and cell states while accounting for brain exposure, compensation, and toxicity.

## Graphical abstract

In gliomas, mitochondria integrate glucose, lipid, protein, and nucleotide metabolism to support proliferation, invasion, treatment resistance, and adaptation, with D‐2‐hydroxyglutarate‐driven remodeling in IDH‐mutant tumors and diverse metabolic states in IDH wild‐type glioblastomas. Effective mitochondrial targeting requires biomarker‐guided strategies that account for blood–brain barrier penetration, intratumoral heterogeneity, metabolic compensation, and toxicity.

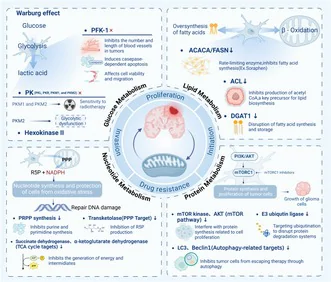

## 1 Introduction

Gliomas are the most common malignant primary tumors of the adult central nervous system (CNS) and have substantial biological and clinical heterogeneity [1, 2]. Current molecular classification separates major diffuse glioma entities by isocitrate dehydrogenase (IDH) status and, for oligodendroglioma, 1p/19q co‐deletion. Glioblastoma (GBM) is predominantly an IDH wild‐type diffuse astrocytic tumor in adults [3, 4]. This distinction is important for metabolic studies, as IDH‐mutant astrocytomas and oligodendrogliomas are exposed to the oncometabolite D‐2‐hydroxyglutarate (D‐2HG), whereas IDH wild‐type GBM evolves under different genetic, microenvironmental, and bioenergetic pressures. GBM remains highly recurrent and lethal despite maximal safe resection, radiotherapy, and alkylating chemotherapy [5, 6, 7, 8]. Targeted therapies and immunotherapies have expanded the experimental landscape but have produced limited benefit regarding unselected GBM populations, emphasizing the need to identify context‐specific biological dependencies [9, 10].

Metabolic reprogramming sustains glioma growth, survival, and resistance to treatment. In addition to ATP production, mitochondria coordinate bioenergetics, redox and calcium homeostasis, reactive oxygen species (ROS) signaling, and biomolecule synthesis [11, 12, 13, 14, 15]. Although the tricarboxylic acid (TCA) cycle and oxidative phosphorylation (OXPHOS) remain central to energy metabolism [16, 17], glioma cells adapt to hypoxia, nutrient limitation, and immune pressure by altering energy production and biosynthesis, often increasing aerobic glycolysis [18]. Such reprogramming is dynamic and state‐dependent, rather than a uniform shift toward glycolysis [18, 19], enabling survival under adverse conditions and promoting tumor progression and treatment resistance [20, 21].

The glioma microenvironment imposes distinct metabolic and pharmacological constraints, including regional hypoxia, fluctuations in oxygen and nutrient availability, and a heterogeneous blood–brain tumor barrier that limits drug exposure [22, 23]. Within individual tumors, proliferative, hypoxic, invasive, and stem‐like populations engage different combinations of glycolysis and mitochondrial respiration [24, 25]. A mitochondria‐enriched subtype of IDH wild‐type GBM exhibits enhanced OXPHOS and selective sensitivity to mitochondrial perturbation, whereas glioma stem‐like cells retain substantial respiratory reserves and adapt their substrate utilization under stress [26, 27]. Beyond ATP production, mitochondria provide citrate, aspartate, one‐carbon units, and redox cofactors for macromolecular synthesis, while coordinating apoptotic and stress signaling. Therefore, the mitochondrial functional state actively determines glioma fitness and therapeutic response, rather than merely reflecting the Warburg effect [12, 13, 14].

This review adopts a mitochondria‐centered framework to integrate glucose, amino acid, protein, lipid, and nucleotide metabolism in gliomas. We distinguished between IDH‐mutant astrocytoma and oligodendroglioma, IDH wild‐type GBM, and glioma stem‐like cell states using supported evidence. Additionally, we examined mitochondrial genome alterations, mitonuclear signaling, and intercellular mitochondrial transfer, followed by a critical evaluation of metabolic therapies and their translational limitations.

## 2 Literature Search Strategy

This narrative review was informed by structured searches of PubMed/MEDLINE, Web of Science, and Scopus for English‐language studies published through August 2026. The search terms combined glioma entities with mitochondrial pathways, genetics, signaling, transfer, and therapy. Priority was given to human tumors, patient‐derived and orthotopic models, and prospective studies; non‐glioma evidence was used only when mechanistically necessary. Duplicate, conference‐only, and irrelevant studies were excluded. No meta‐analysis was performed because the aim was a mechanistic synthesis.

## 3 Mitochondrial Glucose Metabolism Reprogramming in Gliomas

### 3.1 Integrating Glycolysis, TCA Cycle, and OXPHOS

In normal cells, glucose metabolism integrates glycolysis, TCA cycle, and OXPHOS to generate ATP and support biosynthesis. Glycolysis in the cytoplasm converts glucose into pyruvate to produce ATP and NADH [28]. Pyruvate is then decarboxylated in the mitochondria to form acetyl‐CoA, which enters the TCA cycle to generate NADH and FADH2 [29]. These electron carriers fuel OXPHOS, which generates ATP through the electron transport chain (ETC), making it the most efficient ATP‐producing pathway [30]. This integrated metabolic process ensures cellular energy homeostasis and supports vital functions such as cell division and growth [31, 32].

Glucose metabolism in gliomas does not exhibit a uniform Warburg phenotype. Many IDH wild‐type GBMs show increased glycolysis and lactate production, supporting biomass synthesis and an acidic immunosuppressive microenvironment [33, 34, 35]. Nevertheless, the TCA cycle remains important for anaplerosis, redox balance, and lipid, amino acid, and nucleotide synthesis [36, 37, 38]. IDH wild‐type GBM includes an OXPHOS‐enriched mitochondrial subtype, and GSCs can retain greater respiratory reserves than differentiated cells [26, 27]. In IDH1/2‐mutant gliomas, the conversion of alpha‐ketoglutarate (α‐KG) to D‐2HG alters redox and amino‐acid metabolism and inhibits α‐KG‐dependent dioxygenases and transaminases [39, 40, 41]. Therefore, IDH mutations should not be reduced to generalized TCA cycle failure, and metabolic interventions must account for the subtype, cell state, and alternative fuel use [37, 38, 42].

### 3.2 Targeting Key Glycolytic Metabolic Pathways in Gliomas

Glycolytic reprogramming supports glioma growth and treatment adaptation. However, the mitochondrial relevance of individual glycolytic enzymes varies. Hexokinase 2 (HK2) catalyzes the first step of glycolysis and associates with the outer mitochondrial membrane, thereby coupling glucose phosphorylation to mitochondrial bioenergetics and apoptosis regulation [43, 44]. HK2‐dependent glycolysis is enhanced, including IDH‐related experimental contexts, making HK2 a potential therapeutic target [45]. However, evidence from other cancers cannot be directly extrapolated to gliomas. Phosphofructokinase‐1 (PFK‐1) is a cytosolic enzyme that controls glycolytic carbon flow toward pyruvate and lactate; therefore, its contribution to mitochondrial reprogramming is indirect, although disruption of this pathway can exert anti‐glioma effects [46, 47, 48]. Pyruvate kinase M2 (PKM2) catalyzes the final step of glycolysis and promotes glioma growth and metabolic adaptation [49, 50]. PKM2 regulates nutrient and growth factor signaling, promotes tumor cell tumor microenvironment adaptation, and enhances the proliferative capacity. In glioma cells, PKM2 knockdown impairs glycolysis, reducing ATP and pyruvate levels, thereby inhibiting tumor cell proliferation, migration, and invasion [51, 52]. Under oxidative stress, mitochondrial PKM2 can also stabilize BCL2 and suppress apoptosis, providing a direct link between glycolysis and mitochondrial cell‐death signaling [53]. Accordingly, HK2, PFK‐1, and PKM2 should be considered relevant to mitochondrial reprogramming only if they alter mitochondrial substrate supply, redox homeostasis, or apoptosis, rather than being classified as intrinsically mitochondrial enzymes.

As illustrated in Figure 1, glucose metabolism in gliomas is characterized by the Warburg effect, with key enzymes such as HK2, PFK‐1, and PKM2 driving glycolytic flux. Mutations in IDH1/2 contribute to metabolic reprogramming by producing the oncometabolite 2‐HG, which disrupts the TCA cycle and promotes tumor progression. Targeting these metabolic nodes offers promising therapeutic opportunities for treating gliomas.

**FIGURE 1:**
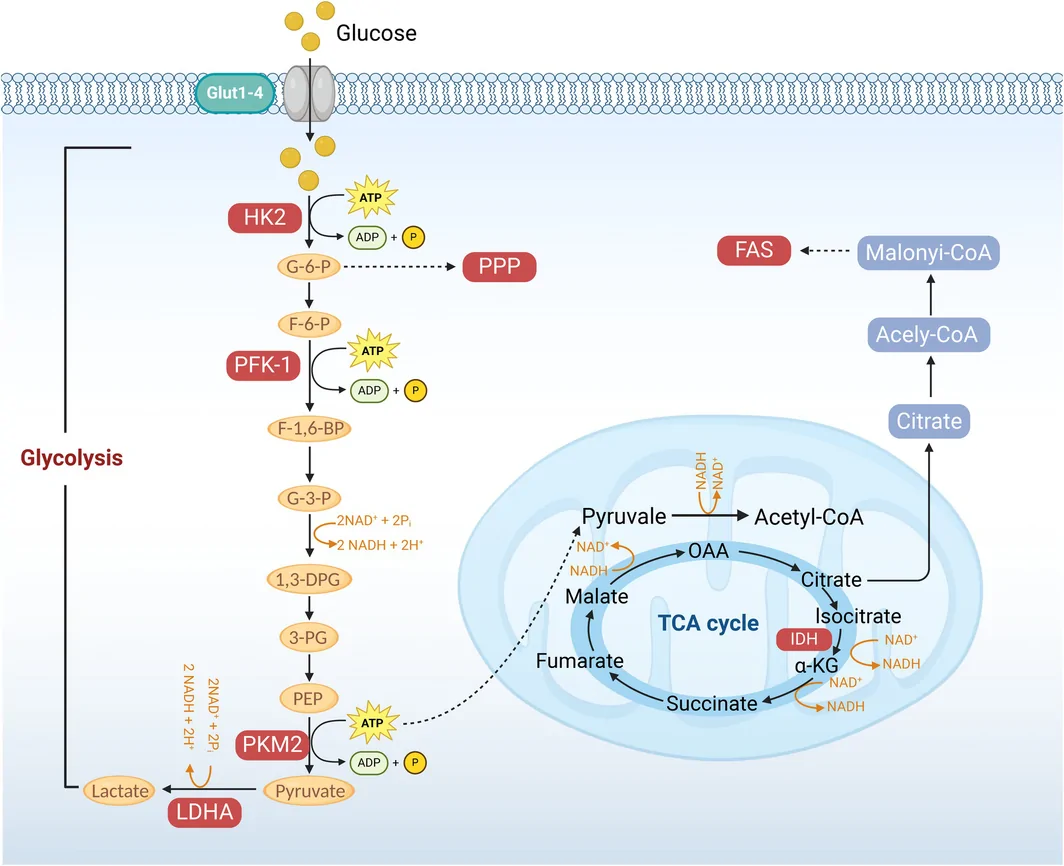
Glucose metabolism and TCA cycle in glioma highlighting key therapeutic targets. This diagram illustrates glucose metabolism and the TCA cycle in glioma, focusing on therapeutic targets HK2, PFK‐1, PKM2, and IDH. Glucose is transported via GLUT1‐4, phosphorylated by HK2, and progresses through glycolysis regulated by PFK‐1 and PKM2 to produce pyruvate. Pyruvate enters the TCA cycle, where IDH catalyzes the conversion of isocitrate to α‐KG. Mutant IDH generates the oncometabolite 2‐HG, driving glioma progression. Targeting HK2, PFK‐1, PKM2, and mutant IDH disrupts key metabolic pathways, offering therapeutic potential in glioma treatment.

### 3.3 Targeting TCA Cycle and OXPHOS Metabolic Pathways in Gliomas

The TCA cycle is a central mitochondrial hub; however, its perturbation has different biological implications in gliomas. IDH1/2 mutations define major adult diffuse glioma subtypes and generate D‐2HG, thereby reshaping epigenetic regulation, redox balance, and metabolic dependencies [39, 54, 55, 56, 57]. In contrast, FH and SDH are established metabolic tumor suppressors in other cancer syndromes, but are not recurrent defining drivers of diffuse glioma. Their relevance is primarily mechanistic, illustrating how altered TCA metabolites can affect cellular signaling rather than supporting their classification as routine therapeutic targets in gliomas [58].

Beyond the TCA cycle, OXPHOS is a potential therapeutic target, and its relevance varies across glioma subtypes and cellular states. Complex I inhibition radiosensitizes glioma cells [59], whereas boxin inhibits mitochondrial ATP synthase and preferentially suppresses primary GBM models [60]. However, respiratory inhibition may also affect non‐malignant cells within the brain microenvironment; for example, Complex I activity contributes to microglial inflammatory function [61]. Mitochondrial dynamic regulators, including DRP1 and MFN1/2, influence cristae organization, respiration, and treatment responses [19, 62, 63]. Therefore, therapeutic intervention should be guided by biomarkers of respiratory dependence or altered mitochondrial dynamics rather than being applied uniformly across gliomas.

### 3.4 Molecular Subtype‐ and Cell‐State‐Specific Mitochondrial Programs

Metabolic analyses should be anchored to contemporary molecular classifications. Although IDH‐mutant astrocytomas, IDH‐mutants, and 1p/19q‐codeleted oligodendrogliomas produce D‐2HG, they do not necessarily share identical downstream metabolic programs. D‐2HG disrupts α‐KG‐dependent enzymes and transaminase activity, creating specific redox and biosynthetic liabilities [6, 41]. One such possibility is de novo pyrimidine synthesis because IDH‐mutant glioma models are selectively sensitive to dihydroorotate dehydrogenase (DHODH) inhibition [64]. The clinical activity of the brain‐penetrant mutant IDH inhibitor vorasidenib in Grade 2 IDH‐mutant gliomas further demonstrated that genotype‐defined metabolic alterations can be therapeutically actionable [65].

IDH wild‐type GBM presents a different challenge owing to its marked metabolic heterogeneity. Pathway‐based profiling identified a mitochondrial subtype enriched in OXPHOS that is sensitive to mitochondrial perturbation, alongside glycolytic and other signaling states [26]. GSCs may also retain substantial respiratory reserves and switch between glycolytic and oxidative metabolism under stressful conditions. These states are further shaped by spatial variations in oxygen and nutrient availability, oncogenic signaling, and treatment exposure [27]. Consequently, inhibiting a single metabolic pathway may eliminate one population while sparing or selecting another. Therefore, studies evaluating mitochondrial targets should report the IDH and 1p/19q status, cell‐state composition, and metabolic phenotype to define the biological context in which dependency occurs.

## 4 Mitochondrial Modulation of Protein Metabolism in Gliomas

### 4.1 Protein Synthesis, Modification, and Degradation in Cellular Homeostasis

Protein homeostasis intersects with mitochondrial biology at several levels. Human mitochondrial DNA encodes only 13 respiratory‐chain polypeptides, and the majority of mitochondrial proteins are nuclear‐encoded, synthesized on cytosolic ribosomes, and imported through mitochondrial translocases [66, 67]. Within the mitochondria, dedicated ribosomes translate mtDNA‐encoded subunits, whereas chaperones, including HSP60/HSP10, support matrix protein folding [68, 69]. This dual genetic origin makes mitochondrial proteostasis intrinsically dependent on coordinated nuclear transcription, cytosolic translation, protein import, and mitochondrial translation rather than on a self‐contained mitochondrial protein‐synthesis system [67, 69].

Post‐translational modifications (PTMs), including phosphorylation and acetylation, regulate respiratory and TCA cycle enzymes, whereas ubiquitin‐dependent quality control, the ubiquitin‐proteasome system (UPS), mitophagy, and the mitochondrial unfolded protein response (UPRmt) limit proteotoxic stress [70, 71, 72, 73, 74, 75]. In gliomas, these processes are particularly relevant because hypoxic and nutrient‐limited niches activate proteostatic stress responses, whereas PI3K/AKT/mTOR and MNK1 signaling sustain cytosolic translation [76]. Thus, the glioma‐specific question is not whether mitochondria perform all cellular protein metabolism but how mitochondrial translation, protein import, quality control, and retrograde stress signaling interact with oncogenic protein synthesis programs. This distinction separates the established mitochondrial mechanisms from the broad concepts of cancer proteostasis.

Disruptions in these processes, particularly mitochondrial dysfunction, contribute to diseases such as cancer and neurodegenerative disorders, emphasizing the importance of mitochondria in maintaining cellular integrity and health.

### 4.2 Protein Synthesis and Potential Therapeutic Targets in Gliomas

Reprogrammed translational control supports glioma growth and metabolic adaptation. PI3K/AKT signaling activates mTORC1, which promotes protein synthesis through 4EBP1 and S6K1 [77, 78]. In a cohort of 132 IDH‐mutant diffuse gliomas, including astrocytomas and 1p/19q‐codeleted oligodendrogliomas, 56.6% showed increased p‐PRAS40, p‐RPS6, and p‐4EBP1 levels, indicating frequent pathway activation [79].

However, effectively inhibiting mTOR remains a challenge. Rapamycin has limited activity because it incompletely suppresses 4EBP1 [80]. The ATP‐competitive inhibitor MLN0128 provides broad pathway inhibition but shows limited in vivo efficacy due to insufficient target residence [81]. RapaLink‐1, a brain‐penetrant bivalent mTOR inhibitor, achieves sustained mTORC1 inhibition and antitumor activity; however, subsequent tumor regrowth suggests that monotherapy is unlikely to produce a durable response [81, 82].

As summarized in Figure 2, glioma proteostasis depends on coordinated protein synthesis and degradation. In addition to PI3K/AKT/mTORC1 signaling, the UPS and autophagy facilitate stress adaptation and treatment resistance, thus representing potential therapeutic targets.

**FIGURE 2:**
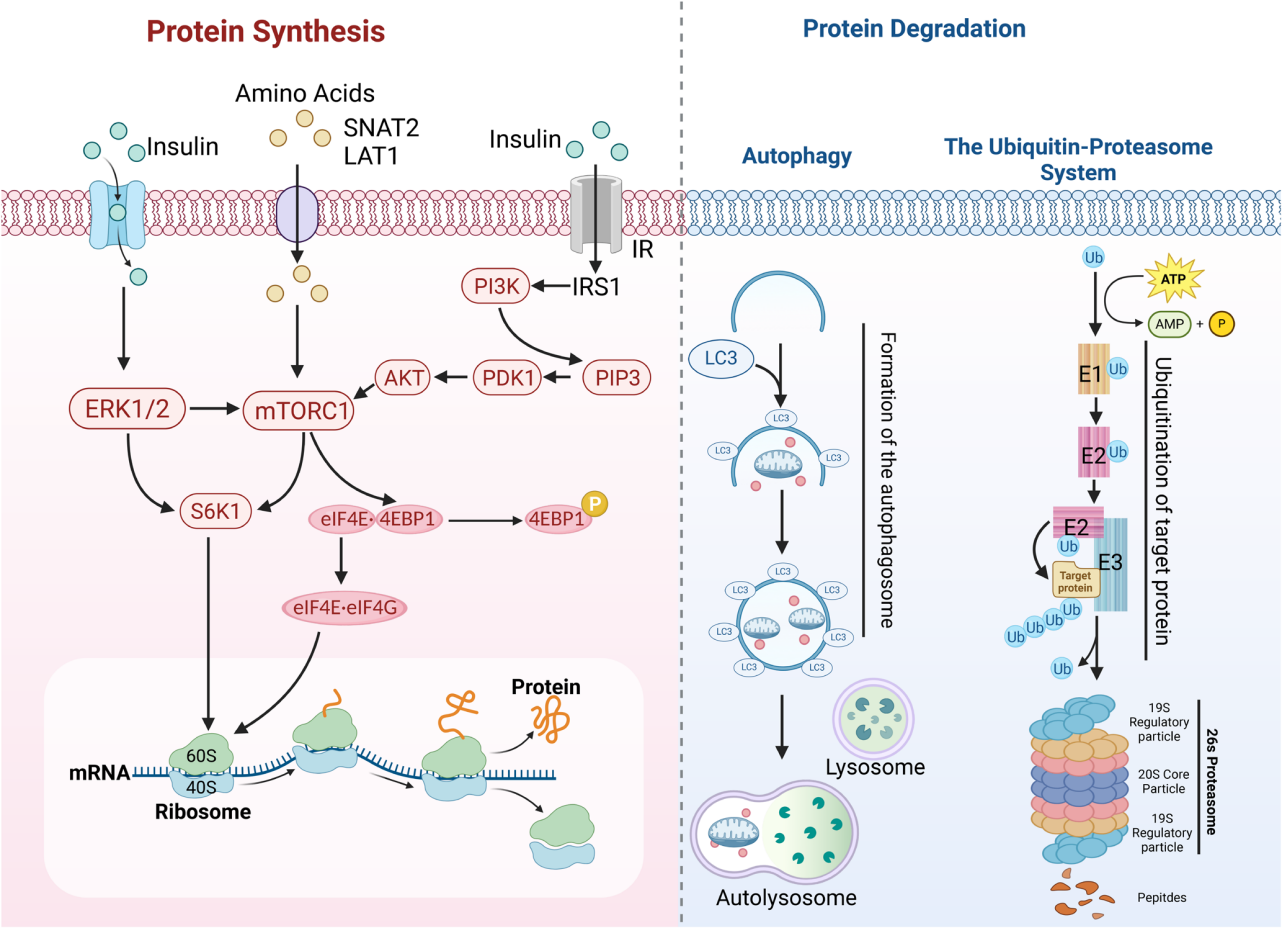
Targetable pathways in protein synthesis and degradation. This figure illustrates key therapeutic targets in protein metabolism, focusing on the mTOR, PI3K/AKT, ubiquitin‐proteasome, and autophagy pathways. Protein synthesis: The PI3K/AKT/mTORC1 axis regulates translation by phosphorylating S6K1 and 4EBP1, promoting ribosomal activity and mRNA translation. mTORC1 is a critical therapeutic target for cancers reliant on increased protein synthesis. Protein degradation: The ubiquitin‐proteasome system tags proteins via E1, E2, and E3 enzymes for degradation by the 26S proteasome, representing a key target for disrupting protein turnover in tumors. Autophagy, initiated by LC3 and autophagosome formation, degrades proteins via lysosomes, providing another target to block tumor cell survival under stress.

### 4.3 PTMs and Corresponding Therapeutic Targets in Gliomas

PTMs influence glioma signaling and metabolic adaptation; however, the strength of glioma‐specific evidence varies across targets. Downstream PI3K/AKT/mTOR phosphorylation regulates translation and growth, whereas acetylation and deacetylation alter mitochondrial and nuclear metabolic programs [83, 84, 85]. Histone deacetylase (HDAC) inhibition provides an instructive translational example: vorinostat showed biological and preclinical activity; however, a Phase I/II study combining vorinostat with temozolomide and radiotherapy in newly diagnosed GBM did not meet its pre‐specified efficacy objective [86]. Thus, HDAC inhibition should be described as an investigative strategy rather than as an established effective glioma therapy. Generally, PTM‐directed agents require evidence that the relevant modification controls glioma‐specific mitochondrial dependency rather than relying on pan‐cancer associations.

### 4.4 Protein Degradation Pathways and Therapeutic Targets in Glioma

Protein degradation and organelle quality control support glioma survival under hypoxic conditions, nutrient limitation, and treatment stress. The UPS controls rapid turnover of damaged or regulatory proteins, whereas autophagy and mitophagy remove macromolecular aggregates and dysfunctional mitochondria [74, 75, 87]. Glioma‐specific studies have shown that interfering with these systems can alter the treatment response and that bortezomib can prime GBM cells, including stem‐like cells, for apoptosis in experimental models; cannabidiol‐induced lethal mitophagy has been demonstrated in glioma cells [88, 89]. In contrast, the core PINK1/Parkin mitophagy mechanism was largely established outside of gliomas [90]; therefore, it provides a mechanistic context rather than direct evidence that PINK1 or Parkin inhibition is an effective glioma therapy. The therapeutic challenge is to identify tumors in which proteasomal or mitochondrial quality control is non‐redundant while avoiding toxicity from broadly suppressing essential protein homeostasis.

## 5 Mitochondrial Lipid Metabolism Reprogramming in Gliomas

### 5.1 Mitochondrial Lipid Metabolism in Normal Conditions and Gliomas

Lipid metabolism connects directly to mitochondria through beta‐oxidation, citrate export, membrane‐lipid remodeling, and redox homeostasis. Cytosolic fatty acid synthesis uses acetyl‐CoA derived from mitochondria‐exported citrate, whereas mitochondrial fatty acid oxidation (FAO) returns acetyl‐CoA to the TCA cycle and generates reducing equivalents for OXPHOS [91, 92]. Mitochondria are also enriched in cardiolipin, a phospholipid required for respiratory chain organization and crista functioning. In contrast, most cholesterol synthesis occurs outside the mitochondria; therefore, mitochondrial cholesterol handling is better considered a specialized aspect of organelle membrane biology than the general mitochondrial cholesterol biosynthesis pathway [93].

Glioma cells exploit lipid synthesis and oxidation in a context‐dependent manner. Glioma‐derived extracellular vesicles can be enriched in FASN, and GBM models show dependence on lipid storage and FAO, which protect cells from lipotoxic and oxidative stress [37, 94, 95]. Mitochondrial citrate supports de novo lipogenesis, whereas FAO supplies respiration when nutrient conditions favor oxidative metabolism. Consequently, the description of mitochondrial dysfunction as uniformly suppressing FAO and forcing gliomas into glycolysis is restrictive. Lipid droplets, DGAT1‐dependent triglyceride storage, FAO, and respiratory capacity form a flexible buffering system that permits survival under fluctuating nutrient and oxygen conditions [37].

### 5.2 Relevant Therapeutic Targets in Lipid Metabolism

Glioma‐specific studies support dysregulated lipid metabolism, although evidence varies among targets. ATP‐citrate lyase (ACLY) generates cytosolic acetyl‐CoA and supports glycolytic metabolism in GBM [96]. ACACA and FASN are established lipogenic enzymes, but evidence linking ACACA to stemness and FASN inhibition to apoptosis is derived mainly from non‐glioma models [97, 98]; therefore, a mechanistic rationale is supported, rather than direct evidence in GBM. In contrast, the FASN inhibitor TVB‐2640 (denifanstat), combined with bevacizumab, has undergone Phase II evaluation for recurrent high‐grade astrocytoma [99, 100].

Gliomas depend on lipid storage and fatty acid oxidation. DGAT1 converts excess fatty acids into triglycerides, and its inhibition increases mitochondrial fatty acid oxidation and ROS accumulation, resulting in mitochondrial damage and apoptosis [37]. Medium‐chain acyl‐CoA dehydrogenase (MCAD) is upregulated in GBM and protects tumor cells by oxidizing potentially toxic medium‐chain fatty acids. MCAD depletion induces lipid accumulation, oxidative stress, and apoptosis [95].

Cardiolipin is required for respiratory chain organization and mitochondrial function. Disrupted cardiolipin metabolism impairs respiratory complexes and OXPHOS, and may contribute to glioma progression [101, 102]. The interaction of cardiolipin with LC3 also links mitochondrial membrane remodeling to mitophagy and apoptosis [103].

As summarized in Figure 3, lipid synthesis, storage, oxidation, and mitochondrial membrane homeostasis collectively support glioma cell survival. ACLY, FASN, DGAT1, and MCAD are potential metabolic targets. However, their therapeutic value requires validation in molecularly defined glioma models and clinical studies.

**FIGURE 3:**
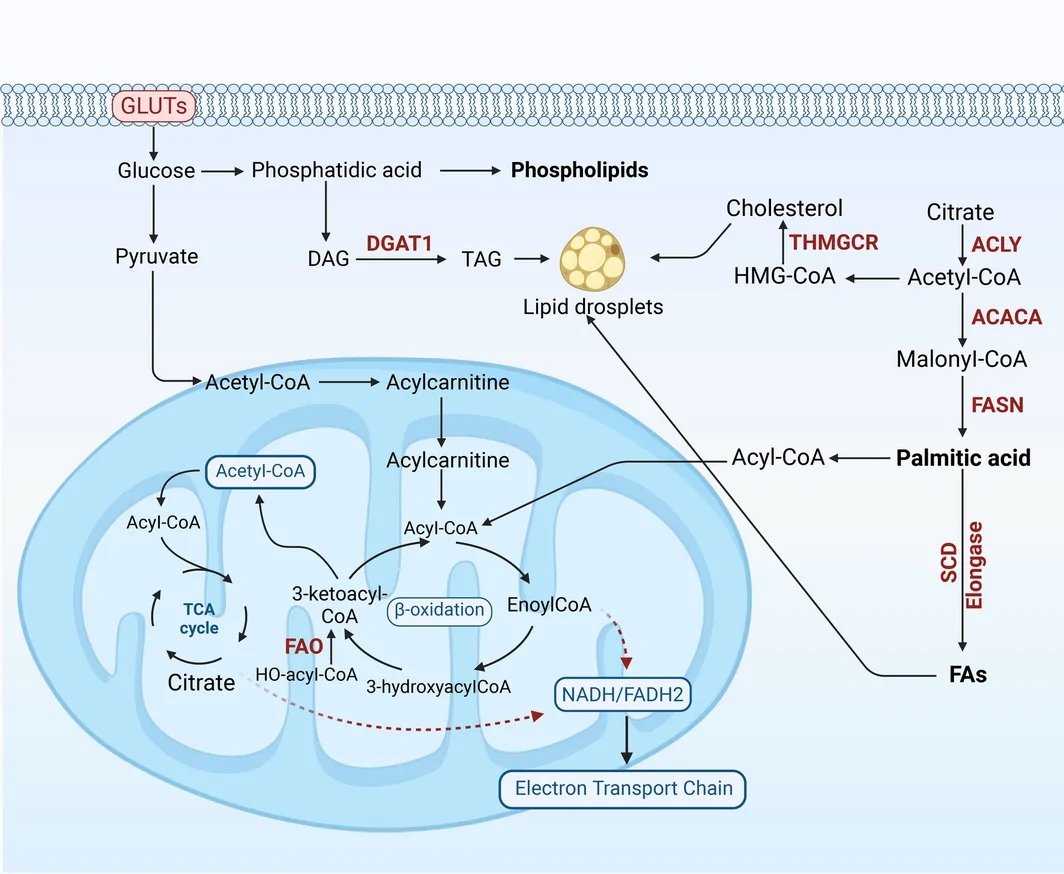
Dysregulated lipid metabolism in glioma and key therapeutic targets. This schematic highlights lipid metabolism in glioma, focusing on therapeutic targets ACACA, FASN, and DGAT1. Citrate from the TCA cycle is converted to acetyl‐CoA by ACLY, fueling fatty acid synthesis. ACACA catalyzes the rate‐limiting step, generating malonyl‐CoA, which is elongated by FASN to form palmitic acid. Fatty acids are further modified or stored as triacylglycerols (TAGs) by DGAT1. Inhibiting ACACA and FASN disrupts fatty acid biosynthesis, while targeting DGAT1 blocks lipid storage, collectively impairing tumor growth and survival.

## 6 Mitochondrial Nucleotide Metabolism Reprogramming in Gliomas

### 6.1 Mitochondrial Nucleotide Metabolism in Physiological Conditions

Nucleotide biosynthesis is compartmentalized, with mitochondria providing essential supporting reactions rather than hosting the complete synthetic pathways. De novo purine synthesis occurs predominantly in the cytosol, but depends on ATP, amino acids, one‐carbon units, and redox cofactors linked to mitochondrial metabolism [104, 105, 106]. A direct mitochondrial connection exists in de novo pyrimidine synthesis, in which DHODH transfers electrons to ubiquinone at the inner mitochondrial membrane, thereby coupling pyrimidine production with the ETC. Mitochondrial respiration also supports aspartate availability, whereas mitochondrial nucleotide pools sustain mtDNA replication and transcription [107, 108, 109].

In proliferating glioma cells, these mitochondrial inputs cooperate with cytosolic pathways. Glycolysis and PPP provide ribose‐5‐phosphate and NADPH, respectively, whereas mitochondrial respiration supports electron transport, aspartate production, and redox homeostasis. Therefore, the mitochondrial contribution to nucleotide metabolism should be understood as the coordination of precursor availability and redox state with cytosolic synthesis, rather than direct control of the entire pathway. This compartmental organization can generate context‐specific metabolic dependencies, including the reliance of IDH‐mutant gliomas on de novo pyrimidine synthesis [64].

### 6.2 Nucleotide Metabolism and Therapeutic Resistance in Glioma

Glioma‐specific studies directly link nucleotide metabolism to the treatment response. In genetically diverse GBM models, elevated purine metabolites are associated with radioresistance, whereas guanine nucleotide synthesis inhibition impairs DNA repair and radiosensitizes orthotopic tumors [110]. Similarly, DHODH inhibition depleted pyrimidine pools, increased DNA damage, suppressed GSC proliferation, and prolonged survival in orthotopic xenograft models [111]. IDH‐mutant gliomas exhibit a particularly strong dependence on de novo pyrimidine synthesis and are sensitive to the brain‐penetrant DHODH inhibitor BAY 2402234 in preclinical models [64]. These observations indicate that nucleotide dependencies vary with tumor genotype and cell state rather than representing a uniform feature of all gliomas.

Although the PPP supplies ribose‐5‐phosphate and NADPH for nucleotide synthesis, it is a cytosolic pathway and should not be classified as part of mitochondrial metabolism. The relevance of the PPP lies in its functional coupling with mitochondrial respiration, precursor availability, and redox regulation [112, 113, 114]. Therefore, therapeutic strategies should prioritize defined dependencies, such as guanine nucleotide synthesis or DHODH, over nonspecific inhibition of the entire PPP or TCA cycle. Measurements of nucleotide pool depletion, DNA damage, and tumor genotype should accompany these interventions to distinguish specific target engagement from generalized metabolic toxicity [64, 110, 111].

Nucleotide metabolism illustrates the functional integration of mitochondrial and cytosolic pathways. Mitochondrial electron transport, aspartate availability, and redox homeostasis cooperate with cytosolic nucleotide synthesis to support nucleic acid production, DNA repair, and treatment resistance in gliomas.

## 7 Mitochondrial Genome Alterations and Communication in Glioma

### 7.1 Mitochondrial DNA and Mitochondrial‐Nuclear Communication

The mitochondrial genome adds a distinct genetic dimension to the glioma metabolism. Primary GBM specimens harbor somatic mtDNA variants that affect respiratory chain genes, including those encoding Complex III and IV subunits [115, 116]. The mtDNA copy number also varies among GBMs and shows age‐dependent associations with clinical outcomes, indicating that it is not a unidirectional marker of tumor aggressiveness [117]. Therefore, respiratory phenotypes should be evaluated in relation to the mtDNA genotype, heteroplasmy, and copy number, together with relevant nuclear alterations.

Mitonuclear communication links mitochondrial stress with nuclear adaptation. Changes in membrane potential, ROS production, NAD+/NADH balance, TCA cycle metabolites, and mitochondrial proteostasis can activate nuclear transcriptional responses that, in turn, regulate mitochondrial biogenesis, protein import, antioxidant defense, and substrate utilization. Relevant examples of gliomas include IDH/D‐2HG‐mediated metabolic–epigenetic coupling, PI3K/AKT/mTOR‐dependent metabolic regulation, and stress‐responsive proteostasis. However, direct causal evidence for canonical retrograde signaling in molecularly defined gliomas remains limited. Therefore, mitonuclear signaling should be regarded as a developing mechanistic framework, rather than a fully characterized glioma pathway.

### 7.2 Intercellular Mitochondrial Transfer

Intercellular mitochondrial transfer extends metabolic adaptations beyond cell‐autonomous mechanisms. In GBM models, astrocyte‐derived mitochondria enter tumor cells through GAP43‐dependent intercellular connections, increasing the respiratory capacity and tumorigenicity [118]. Mitochondrial exchange through the tunneling of nanotube‐like structures can also remodel the metabolism of non‐neoplastic astrocytes under hypoxic conditions [119]. Therefore, the direction and biological consequences of mitochondrial exchange depend on cellular and microenvironmental contexts.

Mitochondrial transfer can be distinguished from intracellular organelle trafficking and EV‐mediated metabolite exchange. Establishing causality requires evidence that donor‐derived mitochondria remain functional in recipient cells, and that selective disruption of transfer alters tumor behavior without broadly impairing cytoskeletal function or cell viability. The GAP43 study provides glioma‐specific support for these criteria and suggests that mitochondrial acquisition may contribute to adaptation or resistance to stress treatments [118].

## 8 Mitochondrial Coordination of the Glioma Metabolic Network

Protein homeostasis and metabolism of glucose, amino acids, lipids, and nucleotides are interconnected via mitochondrial metabolism [31, 34, 67, 104]. Pyruvate‐ and glutamine‐derived carbon replenish the TCA cycle; citrate export supplies cytosolic acetyl‐CoA for lipid synthesis; amino acids provide carbon and nitrogen for anaplerosis and nucleotide production [31, 91, 96]. The ETC maintains a redox balance while supporting aspartate availability and DHODH‐dependent pyrimidine synthesis. The cytosolic PPP complements these processes by supplying ribose‐5‐phosphate and NADPH [107, 108, 109]. Thus, the TCA cycle, ETC, and mitochondrial transport systems integrate carbon flux, redox homeostasis, energy production, and macromolecular synthesis.

This organization also enables metabolic compensation. Glycolytic inhibition may increase reliance on OXPHOS or alternative substrates, whereas lipid storage disruption increases FAO and oxidative stress [25, 26, 32]. IDH mutations illustrate how a single metabolic alteration can affect multiple pathways: D‐2HG modifies epigenetic regulation, transaminase activity, redox homeostasis, and biosynthetic requirements [39, 54, 55, 56, 57]. Therefore, therapeutic strategies should be based on measurable metabolic fluxes and compensatory pathways, rather than the expression of an isolated enzyme.

## 9 Translational Challenges and Limitations of Metabolic Targeting

The clinical application of metabolic therapies is constrained by drug delivery and the therapeutic index. A target may be essential in cultured GBM cells but remain clinically inaccessible if the corresponding inhibitor fails to achieve sustained pharmacologically active concentrations across the heterogeneous blood–brain tumor barrier [23]. Moreover, mitochondrial function is essential in neurons, astrocytes, immune cells, and peripheral tissues, increasing the risk of neurological and systemic toxicity. Therefore, brain penetration, intratumoral pharmacokinetics, target engagement, and normal tissue metabolic reserves should be established before antitumor efficacy is inferred [16, 23, 30].

Intratumoral heterogeneity and metabolic compensation further limit the durability of single‐pathway inhibition. IDH wild‐type GBM contains both glycolytic and mitochondria‐enriched states, whereas GSCs can switch between energy‐producing pathways [24, 25, 26, 27]. Therefore, targeting one pathway may select pre‐existing resistant populations or induce alternative substrate utilization, autophagy, FAO, or redox programs. Although this adaptive capacity provides a rationale for biomarker‐guided combinations, combined metabolic inhibition may narrow the therapeutic window and require pharmacodynamic evidence beyond additive cytotoxicity in vitro.

Clinical studies have illustrated a gap between preclinical activity and patient benefits. Vorinostat combined with standard chemoradiotherapy did not meet its prespecified efficacy objective in newly diagnosed GBM [86], whereas adding everolimus increased severe toxicity without improving progression‐free survival [120]. Denifanstat combined with bevacizumab has shown encouraging Phase II activity in recurrent high‐grade astrocytomas. However, confirmatory evidence is required before FASN inhibition can be considered an established treatment strategy [99]. In contrast, vorasidenib prolongs progression‐free survival in molecularly selected Grade 2 IDH‐mutant gliomas, providing clinical validation for genotype‐directed metabolic therapy [65]. Therefore, future developments should integrate brain exposure, molecular subtypes, metabolic biomarkers, and on‐treatment target engagement.

## 10 Conclusion

Mitochondrial metabolic reprogramming in glioma extends beyond ATP production and cannot be explained by a uniform shift from OXPHOS to glycolysis. By integrating TCA‐cycle flux, electron transport, redox homeostasis, metabolite exchange, and proteostasis, mitochondria connect glucose metabolism with amino acid, lipid, and nucleotide biosynthesis. This framework defines glioma metabolism as a compartmentalized, state‐dependent network and reconciles enhanced glycolysis with persistent mitochondrial dependence.

These dependencies vary across molecular subtypes and cellular states. IDH‐mutant gliomas undergo D‐2‐HG‐dependent metabolic and epigenetic remodeling, whereas IDH‐wild‐type GBM comprises glycolytic and mitochondria‐enriched populations. GSCs further adapt substrate utilization under metabolic and therapeutic stress. Accordingly, metabolic therapies should be guided by molecular subtype and functional phenotype, with confirmation of brain and tumor exposure, target engagement, compensatory responses, and normal‐tissue toxicity. The efficacy of vorasidenib in molecularly selected IDH‐mutant glioma, contrasted with the limited benefit or increased toxicity of unselected strategies in GBM, supports biomarker‐guided metabolic intervention.

Future studies should combine isotope tracing, spatial and single‐cell metabolomics, mitochondrial functional assays, and genomic profiling to identify causal dependencies and track their evolution during treatment and recurrence. The roles of mtDNA alterations, mitonuclear signaling, and intercellular mitochondrial transfer require validation in patient‐derived models, orthotopic systems, and longitudinal clinical specimens. Incorporating pharmacokinetic, pharmacodynamic, and metabolic biomarkers into clinical trials will be essential for defining therapeutic windows and developing rational combination strategies.

## Author Contributions

**Liangqi Jiang:** conceptualization, investigation, data curation, writing – original draft. **Mingrui Li:** investigation, data curation, writing – review and editing. **Zhen Li:** investigation, validation, writing – review and editing. **Qing Liu:** supervision, project administration, writing – review and editing. **Yang Li:** supervision, funding acquisition, writing – review and editing.

## Funding

This work was supported by the National Natural Science Foundation of China (82403075).

## Ethics Statement

As this manuscript is a review article, it does not involve original research with human or animal subjects. Therefore, no ethical approval or statement is required for this submission. We have ensured that all referenced studies are properly cited and conducted in accordance with ethical guidelines.

## Consent

All authors agree to publish.

## Conflicts of Interest

The authors declare no conflicts of interest.

## Data Availability

No data were used to support this study.
